# A Case of Diabetic Chorea Secondary to Appetite Loss Due to COVID-19 Vaccination

**DOI:** 10.7759/cureus.49138

**Published:** 2023-11-20

**Authors:** Hideyuki Inoue, Eiji Kusano, Yasuhiro Shinkai, Hiroyuki Ito

**Affiliations:** 1 Department of Diabetes, Metabolism and Kidney Disease, Edogawa Hospital, Tokyo, JPN; 2 Department of Internal Medicine, Wasshoi Clinic, Tokyo, JPN; 3 Department of Neurology, Edogawa Hospital, Tokyo, JPN

**Keywords:** mrna vaccine, covid-19, involuntary movement, diabetes mellitus, diabetic chorea

## Abstract

A 76-year-old woman with type 2 diabetes mellitus was admitted to our hospital with a complaint of involuntary movements of the limbs and face. Brain MRI demonstrated a bilateral high signal of putamen on the T1 weighted image, and she was diagnosed with diabetic chorea. She took a second dose of the COVID-19 vaccine 28 days before admission and lost her appetite. Consequently, her HbA1c level on admission decreased from 13.5% to 10.0% in 28 days. This case suggests that diabetic chorea could be induced by the rapid amelioration of a hyperglycemic state due to appetite loss after COVID-19 mRNA vaccination.

## Introduction

For more than half a century, it has been reported that diabetic patients with poorly controlled blood glucose levels very rarely present with chorea [1], known as diabetic chorea. With the advancement of diagnostic imaging technologies, it has become clear that the capsular high-signal area on MRI T1-weighted images is a characteristic finding of this disease [2]. The disease concept has been established [3], and detailed clinical features have been clarified. In recent years, several cases of diabetic chorea have been reported due to the rapid correction of hyperglycemia [4-9].

SARS-CoV-2, discovered in Wuhan, China, in December 2019, has spread worldwide at an exceptional rate and poses a significant threat to humanity. Pfizer Inc. and Moderna Inc. in the U.S. developed and launched mRNA vaccines with a completely new mechanism, which have been successful in Japan. However, mRNA vaccines have a higher frequency of local and systemic adverse reactions than conventional vaccines [10,11], which poses a challenge.

We report a case in which a hyperglycemic state was rapidly ameliorated after an adverse reaction caused by inoculation with an mRNA vaccine (mRNA-1273 SARS-CoV-2 Vaccine) manufactured by Takeda/Moderna, and a diabetic chorea was thought to have developed.

## Case presentation

A 76-year-old woman with a 20-year history of type 2 diabetes mellitus was admitted to our hospital due to involuntary movements of the limbs and face. She had Alzheimer’s disease two years before admission. As her dementia worsened, so did her glycemic control, and she was referred to our hospital one year before admission. She was treated with a glucagon-like peptide 1 receptor agonist, an increased insulin dose, and empagliflozin. However, she remained hyperglycemic with an HbA1c of 10% or higher.

She was inoculated with the first dose of mRNA-1273 SARS-CoV-2 vaccine in the right upper arm in 2021. She felt dizzy and fell shortly after receiving a vaccination. After the fall, she could walk without help, but she continued to have mild lightheadedness and loss of appetite.

She visited our department for a routine checkup 35 days after the first vaccination. Her HbA1c was 13.5%, and her postprandial blood glucose was 288 mg/dL. After an outpatient visit, she received the second vaccination at a mass vaccination center on the same day. However, her anorexia worsened the next day.

Fifteen days after the second vaccination, chorea appeared in the right upper limb, spreading to both upper and lower limbs. Then, 26 days after the second vaccination, involuntary movements emerged in the lips. Therefore, she was brought to the hospital by her family and urgently entered 28 days after the second vaccination.

On physical examination, her BMI was 21.9 kg/m^2^. She was clear of consciousness and left-handed. Her blood pressure was 133/65 mmHg, her heart rate was 105 bpm, her body temperature was 36.7 °C, her respiratory rate was 22 beats/min, and her oxygen saturation was 92% (room air). The oral cavity was dry, but there was no prolonged capillary refill time. Dyskinesia of the lips, drooling, and involuntary movements of the extremities were observed. She kept moving her limbs, especially in the right upper extremity, irregularly and repeatedly for short periods. The automatic movements made it difficult to evaluate the tendon reflexes of the upper and lower limbs and ataxia, and walking was impossible. Still, as far as we could observe, there was no motor palsy or sensory disturbance, and the Babinski reflex was bilaterally negative. Involuntary movements were worse during the daytime and during action but not during sleep.

Blood gas analysis showed no metabolic acidosis (Table 1).

**Table 1:** Arterial blood gas on admission pH: potential hydrogen, PaCO_2_: partial pressure of carbon dioxide, PaO_2_: partial pressure of Oxygen, HCO^-^_3_: bicarbonate, AG: anion gap.

Laboratory test	Result value	Reference range
pH	7.42	7.35-7.45
PaCO_2_ (mmHg)	34.8	35-45
PaO_2_ (mmHg)	138	80-100
HCO^-^_3 _(mmol/L)	22.1	22-26
AG (mmol/L)	7.9	10-14

Blood and urine biochemical tests showed a mildly elevated inflammatory response and decreased renal function due to volume depletion. Sodium was 136 mEq/L, chloride was 100 mEq/L, potassium was 4.8 mEq/L, calcium was 8.8 mg/dL, inorganic phosphorus was 4.4 mg/dL, HbA1c was 10.0%, blood glucose was 163 mg/dL, and 24-hour urinary C-peptide was 55.1 μg/day (Table 2).

**Table 2:** Significant lab results on admission CRP: C-reactive protein, BUN: blood urea nitrogen, eGFR: estimated glomerular filtration rate, Cu: copper, HbA1C: hemoglobin A1C.

Laboratory test	Result value	Reference range
CRP (mg/dL)	2.84	≦0.3
Uric acid (mg/dL)	9.5	2.5-7.1
BUN (mg/dL)	38	8-23
Creatinine (mg/dL)	1.63	0.3-1.17
eGFR (mL/min/1.73m^2^)	24	≧60
Sodium (mEq/L)	136	135-147
Chloride (mEq/L)	100	96-108
Potassium (mEq/L)	4.8	3.6-5.0
Calcium (mg/dL)	8.8	8.8-10.2
Inorganic phosphorus (mg/dL)	4.4	2.5-4.5
Glucose (mg/dL)	163	70-110
HbA1c (%)	10.0	4.7-6.2
Urine C-peptide (μg/day)	55.1	29.2-167

**Figure 1:**
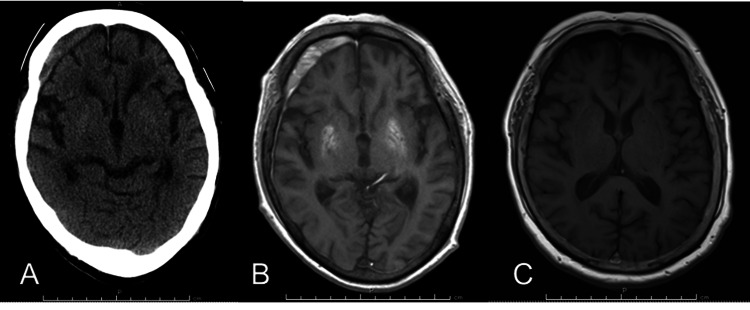
Brain images CT scan of the head at admission showed a subdural hematoma in the right frontal region with no abnormalities in the basal ganglia (A). T1-weighted MRI of the head at the time of admission. High-signal areas were seen in the bilateral putamen (B). T1-weighted MRI image of the head three years before admission. There is no high-signal area in the basal ganglia (C).

Brain CT scan revealed a subdural hematoma in the right frontal region on head CT. Brain MRI T1-weighted images showed high-signal areas in the bilateral putamen. This finding was not seen on a brain MRI image three years earlier (Figure 1A-1C).

SPECT showed extensive hypoperfusion in the cerebral cortex, consistent with the features of Alzheimer's disease.

We diagnosed the patient with diabetic chorea and started treatment with haloperidol 6 mg 1× after supper on the third day. Involuntary movements tended to improve from the next day of treatment and disappeared after about three weeks. In parallel with the treatment of involuntary movements, insulin dosage and diabetes medications were adjusted to improve glycemic control. Finally, six doses of Insulin degludec/liraglutide injected subcutaneously once daily under the medical nutrition therapy based on 1400 kcal/day made a fasting blood glucose of around 100 mg/dL. She could self-inject insulin by herself, but there were some concerns that she might forget, so we decided to have her eldest son or a home care worker watch over her every time she injected insulin. Thus, she was discharged on the 40th hospital day.

## Discussion

Diabetic chorea is involuntary movements that rarely occur in diabetic patients with poor glycemic control, generally characterized by a high signal in the striatum, especially in the putamen on MRI T1-weighted images [3]. The finding in the striatum is contralateral to involuntary movements, indicating that the striatum is involved in the pathogenesis. Although there are few reports of biopsy or autopsy performed at the appropriate time immediately after onset, the leading cause of the disease is considered to be a small infarction of the striatum at this point [12,13].

Other than diabetic chorea, T1-weighted images could show high signal in the striatum in cerebral hemorrhage, genetic disease, metabolic diseases such as Wilson's disease and hepatic encephalopathy, carbon monoxide poisoning, post-cardiac arrest encephalopathy [3]. However, cerebral hemorrhage at the basal ganglion was not seen in the brain CT scan. There was no family history of hereditary diseases such as tuberous sclerosis or neurofibromatosis and no physical and imaging findings suggestive of these diseases. Wilson's disease was unlikely because the low serum ceruloplasmin level, hepatic dysfunction, or corneal abnormalities during ophthalmologic examination was not confirmed. Hepatic encephalopathy was also unlikely because there were no supportive findings for hepatic cirrhosis, such as decreased serum albumin, thrombocytopenia, prolonged prothrombin time, or hepatic atrophy on abdominal CT. No episodes of carbon monoxide poisoning or cardiac arrest were present in this case. In addition, the fact that the patient's symptoms improved with glycemic control and haloperidol after hospitalization also corroborates the diagnosis of diabetic chorea.

Because diabetic chorea is sporadic, its epidemiology has not been fully characterized, and risk factors other than persistent hyperglycemia have yet to be identified. Chua et al. reported that the average blood glucose and glycated hemoglobin levels at the onset of diabetic chorea were 414 mg/dL and 13.1%, respectively [3]. In the present case, HbA1c levels greater than 10% had persisted for 10 months prior to admission, and high blood glucose levels of 400 mg/dL or higher were frequently observed, indicating a chronic hyperglycemic state. In the six months prior to admission, the patient had six outpatient visits, and on four of the six visits, his blood glucose level exceeded 400 mg/dL. The day after the second vaccination, she showed a marked loss of appetite, and 15 days later, involuntary movements appeared, and she was admitted to the hospital 28 days after the second vaccination.

We considered the possibility that the COVID-19 vaccination may have directly influenced the onset of diabetic chorea in this case. However, it was considered unlikely because there were 15 days between the second vaccination and the onset of the disease.

On the other hand, there are several reports that the rapid correction of blood glucose by treatment in diabetic patients with poor glycemic control could trigger the onset of diabetic chorea [4-9]. It is unclear how fast HbA1c declines to cause diabetic chorea. However, Nishio et al. reported the development of diabetic chorea in a 68-year-old male patient with diabetic mellitus when his HbA1c decreased from 14.4% to 10.6% in one month of treatment [6]. In the present case, the diabetes treatment was not intensified. Still, the HbA1c decreased from 13.5% to 10.0% in one month, and rapid correction of hyperglycemia due to anorexia likely triggered diabetic chorea in the present patient.

Delong's hypothesis is often used to explain the mechanism of the onset of chorea and ballism in general. Neural circuits in the basal ganglia are involved in initiating movement and inhibiting unwanted movement. Motor stimuli originating in the cortex are projected to the striatum, the input portion, and reach the internal globus pallidus/substantia nigra pars reticulata (GPi/SNr) by two main pathways; the GPi/SNr serves as the final output portion that inhibits the cortex via the thalamus. One of the two pathways is the direct pathway, in which inhibitory neurons projected from the striatum directly reach the GPi/SNr, where inhibition of the GPi/SNr results in the disinhibition of thalamic neurons. The other is the indirect pathway, in which stimuli projected from the striatum reach the GPi/SNr via the external globus pallidus and subthalamic nucleus (from now on referred to as GPe and STN) and potentiate the action of the GPi/SNr, thereby suppressing thalamic neurons and inhibiting excessive movement. In diabetic chorea, it is thought that the impairment of the indirect pathway, especially the degeneration and loss of inhibitory neurons projecting from the striatum to the GPe, ultimately leads to the disinhibition of thalamic neurons, resulting in abnormal movements such as chorea and ballism [14].

As for the mRNA-1273-SARS-CoV-2 vaccine, local side effects such as pain, redness, swelling, and axillary lymphadenopathy at the vaccination site occurred in 84.2% of patients after the first vaccination. Systemic side effects such as fever, headache, general malaise, myalgia, arthralgia, nausea and vomiting, and chills occurred in 79.4% of cases after the second vaccination [11].

Thus, a high rate of systemic side effects has been shown to occur after the second COVID-19 vaccination. In this case, the anorexia was most likely caused by the vaccination, as the son, who lived with the patient, stated that the anorexia began suddenly the day after the second vaccination.

Furthermore, the COVID-19 mRNA vaccine very rarely causes myocarditis, thrombocytopenic purpura, and various autoimmune diseases such as autoimmune liver disease, Guillain-Barré syndrome, IgA nephropathy, type 1 diabetes, and multiple sclerosis. These adverse reactions are thought to be due to "molecular mimicry" caused by cross-reactions between self-antigens and substances contained in vaccines or synthesized by the body resulting from vaccine reactions [15]. There has been only one report of diabetic chorea after COVID-19 vaccination [16], but a direct causal relationship has not been established. On the other hand, several recent reports have been on developing diabetic chorea during and immediately after treatment for hyperglycemia [4-9]. It is widely accepted that anaerobic metabolism becomes significant in brain tissue under chronic hyperglycemia [3]. The inhibitory neurotransmitter GABA (γ-aminobutyric acid) is utilized as an energy source, resulting in the depletion of GABA, producing chorea [13]. Therefore, the diabetic chorea of our case might be caused by a rapid correction of prolonged hyperglycemia, which was attributed to appetite loss due to the adverse effect of the COVID-19 vaccine.

Hypoglycemia also should be considered one of the possible causes of the onset of this case. It has been reported that various cytokines are induced during hypoglycemia, resulting in vascular endothelial damage and abnormal coagulation due to vascular endothelial damage and platelet function activation and causing cardiovascular events such as cerebral infarction [17]. Although hypoglycemia was not confirmed in this patient, it is quite possible that she was unaware of hypoglycemia while falling asleep at night because her blood glucose fluctuated widely. Indeed, her fasting blood glucose by self-monitoring blood glucose was below 100 mg/dL several times a month. Considering the clinical course with a rapid lift of hyperglycemic state, it is possible that the mechanism described above during hypoglycemia induced transient ischemia of the bilateral basal ganglia, resulting in reversible neuronal damage and the development of chorea.

Diabetic chorea was previously considered predominantly occurring in older women with type 2 diabetes mellitus in the Asian region [18]. Still, recent reports have shown an increasing number of cases from non-Asian regions and a more comprehensive age range of onset [3]. In a disease such as diabetic chorea, which occurs infrequently, reliable epidemiological information may take a long time to accumulate.

## Conclusions

In this study, we experienced a case of diabetic chorea triggered by vaccination with the mRNA-1273 COVID-19 vaccine. Diabetes mellitus is a risk factor for the severity of COVID-19; therefore, vaccination is vital. However, explaining the possibility of sick days due to adverse reactions and reconfirming the measures to be taken during a sick day is essential.
